# Supplementary material for: Effect of Obstructive Sleep Apnea on the Risk of Injuries—A Nationwide Population-Based Cohort Study
Source: Int J Environ Res Public Health. 2021 Dec 20;18(24):13416. doi: 10.3390/ijerph182413416 (PMC8707297; doi:10.3390/ijerph182413416)
Supplement: Supplementary file 1 [file ijerph-18-13416-s001.zip › ijerph-1484266-supplementary.pdf]

Supplementary Table S1. The ICD-9-CM codes used in the study

	ICD-9-CM codes
**Study cohort**	
Obstructive sleep apnea	780.51, 780.53, 780.57;
**Events:** Injury	800-999, E800-E999
Injury diagnosis	
Fracture	800-829
Dislocation	830-839
Sprains and strains	840-849
Intracranial / internal injury	850-869
Open wound	870-899
Injury to blood vessels	900-904
Superficial injury / contusion	910-924
Crushing	925-929
Foreign body entering through orifice	930-939
Burn	940-949
Injury to nerves and spinal cord	950-957
Poisoning	960-989
Others injury	800-999 excluding listed above
Cause of injury	
Unintentional	E800-E949
Traffic	E800-E848
Poisoning (drugs / medicaments / biologicals)	E850-E858
Poisoning (solid and liquid substances / gases / vapors)	E860-E869
Surgical / medical care	E870-E876
Abnormal reaction of medical procedures	E878-E879
Falls	E880-E888
Burns and fires	E890-E899
Environment	E900-E909
Drowning	E910
Suffocation	E911-E915
Other unintentional injuries	E916-E928
Late effects	E929
Adverse drug reaction	E930-E949
Intentional	E950-E969
Suicide	E950-E959
Homicide / Abuse	E960-E969
**Comorbidities**	
Diabetes mellitus	250
Hypertension	401-405
Hyperlipidemia	278

Cerebrovascular disease	410-414
Stroke	430-438
Obesity	278
Anxiety	300.0, 300.2-300.3, 300.9
Depression	296.2-296.3, 300.4, 311

Supplementary Table S2. Baseline characteristics of cohort and comparison cohort in the baseline

**OSA**	**Total**		**With**		**Without**		***P***
**Variables**	**n**	**%**	**n**	**%**	**n**	**%**	
**Total**	34,575		6,915	20.00	27,660	80.00	
**Gender**							0.999
Male	23,200	67.10	4,640	67.10	18,560	67.10	
Female	11,375	32.90	2,275	32.90	9,100	32.90	
**Age (y)**	56.13 ± 17.94		56.11 ± 17.53		56.14 ± 18.04		0.901
**Age group (y)**							0.999
20-44	8,630	24.96	1,726	24.96	6,904	24.96	
45-64	12,180	35.23	2,436	35.23	9,744	35.23	
≧65	13,765	39.81	2,753	39.81	11,012	39.81	
**DM**							0.983
Without	28,728	83.09	5,745	83.08	22,983	83.09	
With	5,847	16.91	1,170	16.92	4,677	16.91	
**HTN**							0.947
Without	31,492	91.08	6,297	91.06	25,195	91.09	
With	3,083	8.92	618	8.94	2,465	8.91	
**Hyperlipidemia**							0.946
Without	34,233	99.01	6,846	99.00	27,387	99.01	
With	342	0.99	69	1.00	273	0.99	
**CVD**							0.980
Without	30,643	88.63	6,128	88.62	24,515	88.63	
With	3,932	11.37	787	11.38	3,145	11.37	
**Stroke**							0.987
Without	30,423	87.99	6,085	88.00	24,338	87.99	
With	4,152	12.01	830	12.00	3,322	12.01	
**Obesity**							0.799
Without	34,479	99.72	6,895	99.71	27,584	99.73	
With	96	0.28	20	0.29	76	0.27	
**Anxiety**							0.899
Without	34,186	98.87	6,836	98.86	27,350	98.88	
With	389	1.13	79	1.14	310	1.12	
**Depression**							0.959
Without	33,972	98.26	6,794	98.25	27,178	98.26	

With	603	1.74	121	1.75	482	1.74

P: Chi-square / Fisher exact test on category variables and t-test on continue variables.

Abbreviations: OSA = obstructive sleep apnea; y = years; DM = diabetes mellitus; HTN = hypertension; CVD = cardiovascular disease.

Table S3. Years of follow-up

OSAs	Min	Median	Max	Mean ± SD
With	0.01	5.64	15.88	10.29 ± 10.76
Without	0.01	6.97	15.98	10.94 ± 11.29
Total	0.01	6.82	15.98	10.81 ± 10.83

Table S4. Years to injury

OSAs	Min	Median	Max	Mean ± SD
With	0.01	4.46	15.54	5.54 ± 4.22
Without	0.01	4.80	15.79	5.63 ± 4.33
Total	0.01	4.53	15.79	5.61 ± 4.31
